# Sickle Cell and Malaria

**DOI:** 10.1371/journal.pmed.0020150

**Published:** 2005-05-31

There are at least 300 million acute cases of malaria each year globally, resulting in more than a million deaths. Ninety percent of deaths due to malaria occur in Africa south of the Sahara, and most occur in young children. Of the four types of human malaria—Plasmodium vivax, P. malariae, P. ovale, and P. falciparum—P. falciparum malaria is most common in Africa, and it accounts for most of the extremely high mortality south of the Sahara.

Malaria parasites are developing unacceptable levels of drug resistance, and many insecticides are no longer useful against mosquitoes transmitting the disease. Vaccine research has produced few hopeful candidates, and although millions of dollars are poured into research, an effective vaccine is years away.

One recurring theme in malaria vaccine research has been the high frequency of the gene for sickle cell hemoglobin (HbS) in malaria endemic regions, which is believed to be due to a heterozygote (HbAS) advantage against fatal malaria. The mechanism behind the high degree of resistance conferred by HbAS in severe and complicated malaria is still unknown, but recent observations have suggested the mechanism might involve an immune component.

In this month's *PLoS Medicine*, Thomas Williams and colleagues reason that the best way to test whether malaria protection by HbAS has a significant immune component is to see whether protection varies with age. They studied the age-specific malaria pattern in 1,054 children and adults living in Kilifi District on the coast of Kenya. They argued that if the malaria protection provided by HbAS were innate, it should be independent of malaria exposure and remain constant with age. However, if immune mechanisms were involved, protection should increase with age until children become functionally immune, when additional immunological advantage should be lost.

They found that overall HbAS was nearly 40% protective against mild clinical malaria. Protection varied with age, increasing from 20% to 60% during the first ten years of life, and thereafter returning to 30% in children more than ten years old.

The authors admit that this observation could be due to any factor that affects malaria risk and varies with age but state that accelerated immune acquisition seems the most likely explanation. They suggest several mechanisms for how HbAS could accelerate immune acquisition; for example, immunity could be mediated by accelerated acquisition of antibodies to altered host antigens expressed on the parasite-infected red cell surface.[Fig pmed-0020150-g001]

**Figure pmed-0020150-g001:**
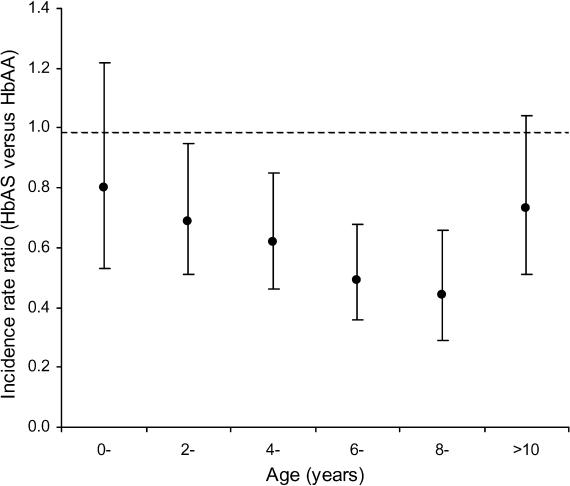
Incidence rate ratio for malaria in HbAS versus HbAA children by age and genotypic group

In discussing their findings the authors point out that their study focused on mild malaria. For accelerated malaria-specific immunity to be relevant to HbAS selection it would have to operate within a period of maximum risk for severe and fatal malaria. They note that in a recent study, conducted by another group in western Kenya, protection against severe malaria by HbAS was only seen in children 2–16 months old, but that in that study, no analysis was presented that addressed the effect of age within that range.

The authors conclude that the relevance of their current observations on mild clinical malaria to protection against severe and fatal malaria are unknown, and that further work must be done to better understand the role of HbAS in protection against malaria.

